# Triglyceride-glucose index is associated with heart failure with preserved ejection fraction in different metabolic states in patients with coronary heart disease

**DOI:** 10.3389/fendo.2024.1447072

**Published:** 2024-11-04

**Authors:** Zhu Li, Xiang Fan, Yijia Liu, Lu Yu, Yuanyuan He, Lin Li, Shan Gao, Wei Chen, Rongrong Yang, Chunquan Yu

**Affiliations:** ^1^ School of Basic Medical Sciences, Zhejiang Chinese Medical University, Hangzhou, China; ^2^ Second Teaching Hospital of Tianjin University of Traditional Chinese Medicine, Tianjin, China; ^3^ Tianjin University of Traditional Chinese Medicine, Tianjin, China; ^4^ Department of Molecular Imaging and Nuclear Medicine, Tianjin Medical University Cancer Institute and Hospital, Tianjin, China; ^5^ National Clinical Research Center for Cancer, Tianjin Key Laboratory of Cancer Prevention and Therapy, Tianjin’s Clinical Research Center for China, Tianjin, China

**Keywords:** triglyceride-glucose index, coronary heart disease, heart failure, heart failure with preserved ejection fraction, metabolic states

## Abstract

**Background:**

The triglyceride-glucose (TyG) index is a surrogate indicator of insulin resistance. Therefore, we aimed to determine the association between TyG index and heart failure (HF) with preserved ejection fraction (HFpEF) in patients with coronary heart disease (CHD) and to explore whether such associations would be modified by different metabolic states.

**Methods:**

Among 107,301 CHD patients, 62,794 were included to analyze the relationship between the TyG index and HF. Among them, 8,606 patients who had undergone echocardiography were included to identify different types of HF, including HF with reduced ejection fraction (HFrEF), HF with intermediate-range ejection fraction (HFmrEF), and HFpEF. Among them, 1896 patients were diagnosed with HFpEF. Logistic regression was used to analyze the relationship between the TyG index and HFpEF in CHD patients. In addition, the association between TyG index and HFpEF according to sex, age, blood lipids, and blood pressure was assessed.

**Results:**

A baseline analysis of CHD patients divided into four groups according to the tertile level of the TyG index showed significant differences in the related parameters between the groups. In the multi-adjusted models, the TyG index was significantly associated with the risk of HFpEF (odds ratio [OR]: 1.17; 95% confidence interval [CI]: 1.09–1.25). After adjustment for multivariates, TyG index levels for T2 (OR: 1.33; 95% CI: 1.16–1.52) and T3 (OR: 1.52; 95% CI: 1.32–1.74) were associated with increased OR in HFpEF. In addition, the TyG index of CHD patients was significantly associated with HFpEF in older adults aged > 60 years (OR: 1.20; 95% CI: 1.11–1.29), hypertension (OR: 1.27; 95% CI: 1.17–1.37), and dyslipidemia (OR: 1.15; 95% CI: 1.08–1.24). Moreover, the OR (OR: 1.23; 95% CI: 1.11–1.36) in women is higher than in men (OR: 1.17; 95% CI: 1.02–1.22, indicating a stronger association between TyG index and HFpEF in women.

**Conclusions:**

Our findings demonstrated a significant association between TyG index and HFpEF in CHD patients. Furthermore, TyG index was independently associated with HFpEF in hypertension, dyslipidemia, and older patients (aged > 60 years). In addition, the association between the TyG index and HFpEF in CHD patients differed according to sex.

## Graphical Abstract

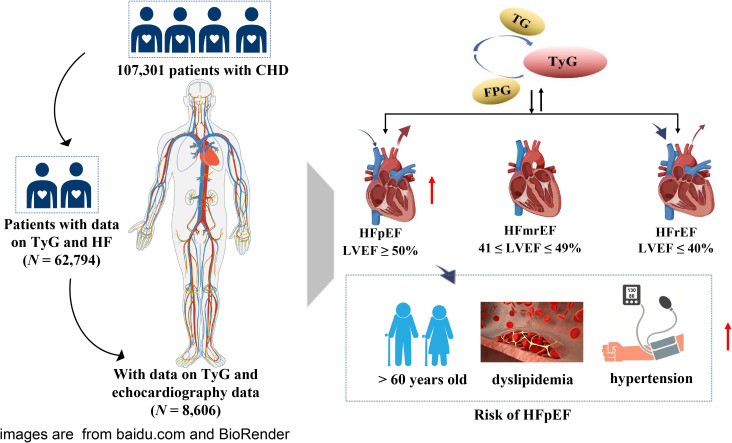

## 1 Introduction

Coronary heart disease (CHD) is an atherosclerotic disease. Its pathological process leads to coronary artery stenosis, which in turn leads to myocardial ischemia, myocardial necrosis, myocardial systolic dysfunction, and heart failure (HF) due to decreased ejection capacity (1, 2). HF with preserved ejection fraction (HFpEF) is the most common type of HF, diagnosed in approximately 50% of HF patients (3). The American College of Cardiology/American Heart Association Task Force on Clinical Practice Guidelines and the Heart Failure Society of America (4) reported an annual increase in the percentage of hospitalizations due to HFpEF in patients with HF. By 2020, left ventricle ejection fraction (LVEF) exceeding 40% is anticipated in 65% of hospitalized patients with HF. HFpEF is associated with high morbidity, readmission rates, and readmission mortality, therefore, its prevention and treatment require further investigation.

The triglyceride-glucose (TyG) index is used as a marker of insulin resistance (IR), which is implicated in the development of non-communicable diseases (5, 6). The TyG index is associated with a high prevalence of coronary artery disease and an increased risk of major adverse cardiovascular and cerebrovascular events (7–11). TyG index and carotid plaque demonstrated a significant association in CHD patients (12). A Mendelian randomization study showed that the TyG index can be used as a more sensitive pre-diagnostic indicator of cardiovascular disease, which could provide a quantitative risk for cardiometabolic outcomes, including HF (13). The TyG index is a novel biomarker of myocardial fibrosis in HF patients and can be considered a useful risk stratification metric for the management of HF (14). However, no relevant studies have investigated the relationship between the TyG index and HF or the types of HF in CHD patients, especially HFpEF.

Therefore, this study aimed to clarify the association between the TyG index and HF in CHD patients and investigate the association between the TyG index and different types of HF, especially HFpEF. Identifying simpler biochemical indicators to prevent the risk of HF may aid in the clinical management of CHD.

## 2 Methods

### 2.1 Patients

**Figure 1:**
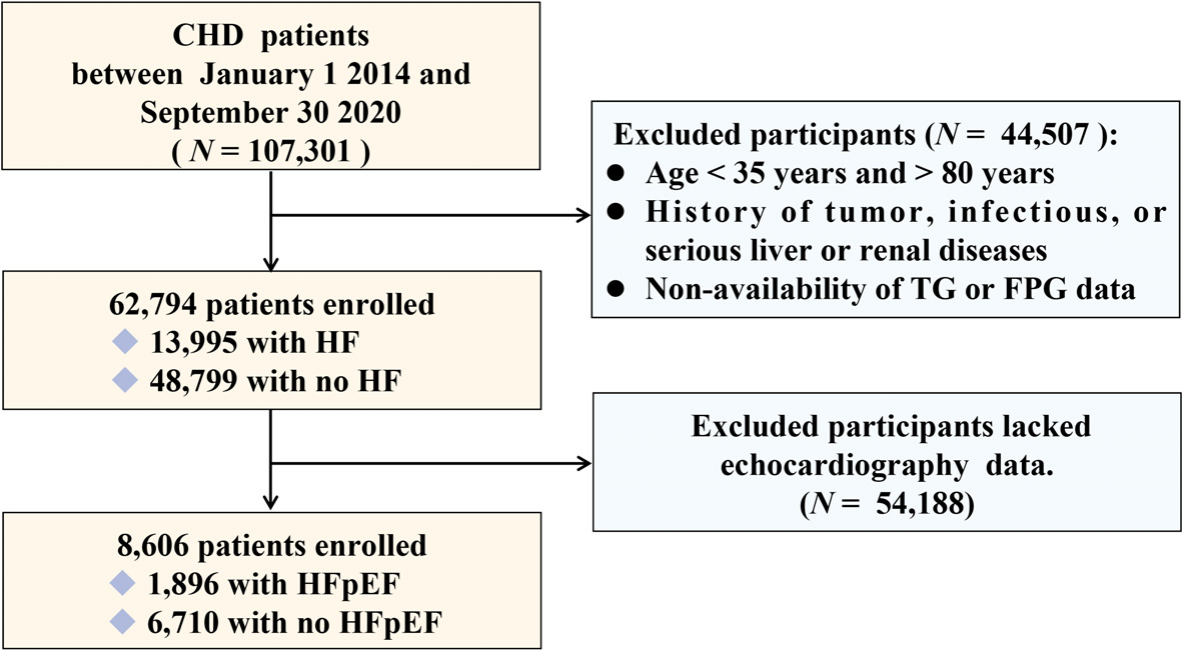
Flow chart of patient selection.

This was a large-scale, multicenter, retrospective, cross-sectional study. Between January 1, 2014, and September 30, 2020, 107,301 CHD patients admitted to six hospitals in Tianjin were included. Following the exclusion of patients aged < 35 years or > 80 years, those with tumor, infectious, or severe liver or kidney diseases, and patients lacking data on triglycerides (TGs) and fasting plasma glucose (FPG),62,794 participants were included in the study. Among them, 8,606 patients who had undergone echocardiography were included to identify different types of HF, including HF with reduced ejection fraction (HFrEF), HF with intermediate-range ejection fraction (HFmrEF), and HFpEF. A flowchart of patient selection is shown in **Figure 1**. This study was approved by the ethics committee of the Tianjin University of Traditional Chinese Medicine (TJUTCM-EC20190008) and registered with the Chinese Clinical Trial Registry (ChiCTR-1900024535) and ClinicalTrials.gov (NCT04026724).

### 2.2 Data collection

Age, sex, smoking, alcohol consumption, and medication history were obtained through standard structured questionnaires (15, 16). Fasting venous blood samples were collected from all the participants early in the morning. FPG, total cholesterol (TC), high-density lipoprotein cholesterol (HDL-C), TG, low-density lipoprotein cholesterol (LDL-C), glycated hemoglobin (HbA1c), uric acid, urea, and creatinine levels were measured using an automatic hematology analyzer. Standard laboratory procedures for quality control were strictly followed. The TyG index was calculated as follows: Ln[TG (mg/dL) × FPG (mg/dL)/2] (17). The hyperlipidemia status of the participants was evaluated based on the National Cholesterol Education Program. Hyperlipidemia was defined as having a TG  ≥ 150 mg/dL (1.7 mmol/L), TC ≥ 200 mg/dL (5.18 mmol/L), LDL-C ≥ 130 mg/dL (3.37 mmol/L), or HDL-C ≤ 50 mg/dL (1.30 mmol/L) in women and ≤ 40 mg/dL (1.04 mmol/L) in men. Moreover, participants who reported using lipid-lowering medications were considered to have hyperlipidemia. Participants meeting any one of the aforementioned criteria were diagnosed to have hyperlipidemia (18, 19). Systolic blood pressure (SBP) and diastolic blood pressure (DBP) were measured by experienced technicians at heart level using automatic blood pressure monitors. Hypertension was defined as having a SBP ≥ 130 mmHg or a DBP ≥ 80 mmHg (20).

CHD was defined according to the International Classification of Diseases 10^th^ revision, primary care health records, and the American College of Cardiology Foundation/American Heart Association criteria for HF. HF is usually diagnosed based on clinical symptoms, physical examination findings, laboratory tests, and imaging studies. Detailed records are available in the RCSCD-TCM database. HF included congestive HF, left ventricular failure, New York Heart Association (NYHA) Heart Function class II–IV (21), and unspecified HF. Different types of HF use the left ventricular ejection fraction (LVEF) measured using echocardiography as a cut-off for the inclusion/exclusion criteria. The European Society of Cardiology Guidelines (22) classified HF patients into the following three groups/categories based on LVEF: HFpEF, patients with LVEF ≥ 50%; HFmrEF, patients with 41 ≤ LVEF ≤ 49%; and HFrEF, patients with LVEF ≤ 40%.

### 2.3 Statistical analyses

The characteristics of participants in the different groups were compared using *χ^2^* tests for categorical variables, and Mann–Whitney U test and Kruskal–Wallis H test for continuous variables. Odds ratios (ORs) and 95% confidence intervals (CIs) of HFpEF were estimated for the TyG index using logistic regression. To further explore the potential nonlinear association between the TyG index and HFpEF, restricted cubic spline (RCS) regression model with four knots was used. The collinearity of the different models was tested before logistic regression. Sex, age, smoking, alcohol consumption, SBP, DBP, TG, HDL-C, uric acid, urea, creatinine, hypertension, hyperlipidemia, and use of antihypertensives, antilipidemic drugs, and antiplatelets were considered as potential confounders in this association. Missing values were imputed using the multiple imputation method. All statistical analyses were performed using SPSS 24.0 (IBM Corp., Armonk, NY, USA).

## 3 Results

### 3.1 Baseline characteristics

Baseline analysis revealed significant differences in related parameters among 62,794 CHD patients with and without HF (*P* < 0.001) (**Supplementary Table S1**). Among them, 1896 out of 8,606 patients who had undergone echocardiography were diagnosed with HFpEF. The average age of the participants was 65 years, and the proportion of men (56.8%) was higher (**Table 1**). The patients were divided into three groups according to the tertile level of the TyG index (T1: TyG index < 10.09, T2: 10.09 ≤ TyG index ≤ 10.71, T3: TyG index > 10.71). In general, FPG, TC, TG, LDL-C, HbA1c, urea, the proportion of HFrEF, HFmrEF, HFpEF, hyperlipidemia, and use of antihypertensives, antilipidemic drugs, and antiplatelets were positively associated with the tertile level of the TyG index, whereas HDL-C and smoking and alcohol consumption were negatively associated with the tertile level of the TyG index (**Supplementary Table S2**).

**Table 1:** General characteristics of the study participants according to HFpEF.

Characteristic	Total (*n*=8606)	No HFpEF (*n*=6710)	HFpEF (*n*=1896)	*P*-value
Sex, n (%)				<0.001
Male	4891 (56.8)	3886 (57.9)	1005 (53.0)	
Female	3715 (43.2)	2824 (42.1)	891 (47.0)	
Age, years, median (IQR)	64 (58-69)	63 (58-68)	65 (60-70)	<0.001
SBP, mmHg, median (IQR)	140 (125-157)	140 (125-155)	141 (130-160)	<0.001
DBP, mmHg, median (IQR)	80 (80-90)	80 (80-90)	80 (77-90)	0.311
TyG index	10.34 (9.98-10.98)	10.30 (9.96-10.95)	10.46 (10.04-11.09)	<0.001
FPG, mmol/L, median (IQR)	6.22 (5.20-8.59)	6.13 (5.15-8.45)	6.63 (5.36-9.05)	<0.001
HbA1c, %, median (IQR)	6.50 (5.70-7.96)	6.50 (5.70-8.00)	6.45 (5.70-7.80)	0.431
LDL-C, mmol/L, median (IQR)	2.82 (2.17-3.52)	2.85 (2.19-3.54)	2.75 (2.11-3.44)	0.013
HDL-C, mmol/L, median (IQR)	1.02 (0.85-1.24)	1.02 (0.86-1.24)	1.02 (0.84-1.24)	0.270
TG, mmol/L, median (IQR)	1.38 (1.00-1.97)	1.38 (1.00-1.97)	1.37 (0.99-1.98)	0.407
TC, mmol/L, median (IQR)	4.47 (3.68-5.33)	4.50 (3.71-5.36)	4.34 (3.58-5.16)	<0.001
Uric Acid, μmol/L, median (IQR)	317 (255-391)	314 (254-385)	332 (261-413)	<0.001
Urea, μmol/L, median (IQR)	5.63 (4.54-7.10)	5.53 (4.50-6.90)	6.01 (4.74-8.28)	<0.001
Creatinine, μmol/L, median (IQR)	70.90 (58.60-86.70)	69.80 (57.90-84.40)	75.35 (61.60-98.38)	<0.001
Smoking, n (%)	3088 (35.9)	2489 (37.1)	599 (31.6)	<0.001
Drinking, n (%)	7410 (86.1)	5808 (86.6)	1602 (84.5)	0.022
LVEF,%, median (IQR)	62 (59-65)	62 (59-65)	61 (58-64)	<0.001
Glucose regulation state, n (%)				<0.001
Normal glucose regulation	3117 (36.2)	2554 (38.1)	563 (29.7)	
Prediabetes	2143 (24.9)	1646 (24.5)	497 (26.2)	
Diabetes	3346 (38.9)	2510 (37.4)	836 (44.1)	
Hypertension, n (%)	6486 (75.37)	5024 (74.87)	1462 (77.11)	0.050
Dyslipidemia, n (%)	7268 (84.45)	5732 (85.42)	1536 (81.01)	<0.001
Hypertension and dyslipidemia, n (%)	5560 (64.7)	1203 (63.4)	4364 (65.0)	<0.001
Use of antihypertensives, n (%)	6331 (73.6)	6273 (93.5)	1774 (93.6)	0.903
Use of antilipidemic, n (%)	5450 (63.3)	4225 (63.0)	1225 (64.6)	0.190
Use of antiplatelets, n (%)	4564 (53.0)	3458 (51.5)	1106 (58.3)	<0.001

Data are presented as median (interquartile range) or number (proportion, %).

TyG, triglyceride-glucose index; SBP, systolic blood pressure; DBP, diastolic blood pressure; FPG, fasting plasma glucose; TC, total cholesterol; TG, triglycerides; HDL-C, high-density lipoprotein cholesterol; LDL-C, low-density lipoprotein cholesterol; HbA1c, glycated hemoglobin; IQR, interquartile range; LVEF, left ventricular ejection fraction; HF, heart failure; HFrEF, HF with reduced ejection fraction; HFmrEF, HF with intermediate-range ejection fraction; HFpEF, HF with preserved ejection fraction; NYHA, New York Heart Association.

### 3.2 Association between TyG index and the risk of HFpEF

**Figure 2:**
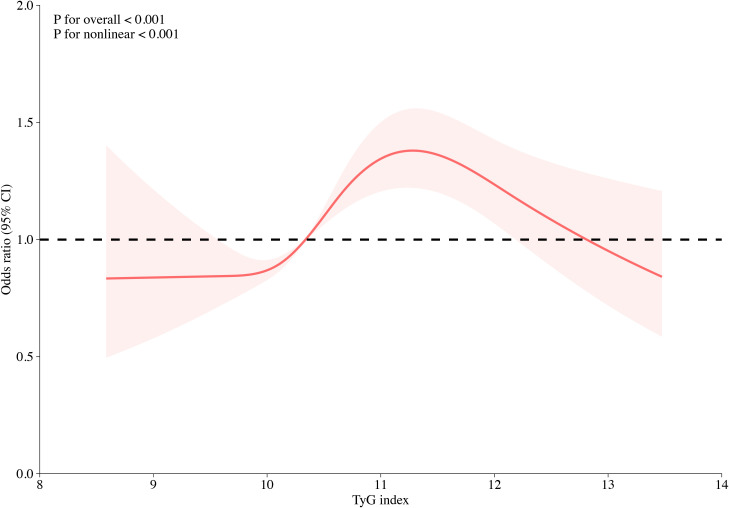
Multivariable-adjusted odds ratios for HFpEF in CHD patients based on restricted cubic spines for the TyG index. Red lines indicate references for hazard ratios, and red areas represent 95% confidence intervals. The model was adjusted for sex, age, smoking, alcohol consumption, SBP, DBP, TG, HDL-C, uric acid, urea, creatinine, hypertension, hyperlipidemia, and use of antihypertensives, antilipidemic agents, and antiplatelets.

Multivariate logistic regression analysis revealed that TG, FPG, and TyG index were significantly associated with the risk of HF, of which TyG index had the highest OR value (OR: 1.14; 95% CI: 1.06–1.22) (**Supplementary Table S3**). Logistic regression models were constructed to show that the TyG index was significantly associated with HFpEF before and after multivariate adjustment (*P* < 0.001) (**Table 2**). When the TyG index was analyzed as a continuous variable, it was significantly associated with HFpEF (OR: 1.17; 95% CI: 1.09–1.25). When the TyG index was considered a classified variable, the risk of HFpEF for patients in T2 and T3 was 1.33 and 1.52 times higher, respectively, than the risk for patients in T1. The associations between the univariate analysis and risk of HFpEF are analyzed in detail in **Supplementary Table S4**. The association between TyG index and the risk of different types of HF, including HFrEF, HFmrEF, and HFpEF, was further evaluated. The results indicated that the associations remained significantly different (**Supplementary Table S5**). The restricted cubic spline models showed that the risk of HFpEF initially remained stable, followed by a rapid increase, and a rapid decrease (**Figure 2**).

**Table 2:** Association between TyG index and the risk of HFpEF.

Variables	HFpEF			
	OR (95% CI)^a^	*P-*value	OR (95% CI)^b^	*P-*value
TyG index	1.17 (1.10–1.24)	< 0.001	1.17 (1.09–1.25)	< 0.001
T1	Reference		Reference	
T2	1.30 (1.14–1.47)	< 0.001	1.33 (1.16–1.52)	< 0.001
T3	1.50 (1.32–1.71)	< 0.001	1.52 (1.32–1.74)	< 0.001
*P-*trend		< 0.001		< 0.001

T1: TyG index < 10.09, T2: 10.09 ≤ TyG index ≤ 10.71, T3: TyG index > 10.71.

a Model 1: adjusted for sex and age.

b Model 2: adjusted for sex, age, smoking, alcohol consumption, SBP, DBP, TG, HDL-C, uric acid, urea, creatinine, hypertension, hyperlipidemia, and use of antihypertensives, antilipidemic drugs, and antiplatelets.

TyG, triglyceride-glucose index; HFpEF, heart failure with preserved ejection fraction; OR, odds ratio; CI, confidence interval.

### 3.3 Association between TyG index and the risk of HFpEF according to sex and age

Association between the TyG index and the risk of HFpEF according to age and sex are summarized in **Table 3**. After multivariate adjustment, the TyG index of CHD patients was significantly associated with HFpEF in older patients aged > 60 years (OR: 1.20; 95% CI: 1.11–1.29). Multivariate logistic regression analysis showed that the TyG index levels for T2 and T3 were associated with an increased OR for HFpEF when T1 was used as a reference, with the highest association observed for T3 in older adults (OR: 1.56; 95% CI: 1.33–1.82). However, no independent association was observed in middle-aged patients aged ≤ 60 years (*P* > 0.05).

**Table 3:** Association between TyG index and the risk of HFpEF according to age and sex.

Group		Variables	HFpEF			
			OR (95% CI)^a^	*P*-value	OR (95% CI)^b^	*P*-value
Age	≤ 60 years	TyG index	1.06 (0.94–1.20)	0.320	1.09 (0.96–1.25)	0.168
		T1	Reference		Reference	
		T2	1.31 (1.01–1.69)	0.045	1.40 (1.05–1.85)	0.020
		T3	1.26 (0.98–1.63)	0.072	1.40 (1.05–1.85)	0.021
		*P*-trend		0.083		0.024
	> 60 years	TyG index	1.21 (1.12–1.30)	< 0.001	1.20 (1.11–1.29)	< 0.001
		T1	Reference		Reference	
		T2	1.29 (1.11–1.49)	0.001	1.30 (1.12–1.52)	0.001
		T3	1.59 (1.37–1.84)	< 0.001	1.56 (1.33–1.82)	< 0.001
		*P*-trend		< 0.001		< 0.001
*P*-interaction						< 0.001
Sex	Male	TyG index	1.13 (1.04–1.22)	0.005	1.17 (1.02–1.22)	0.015
		T1	Reference		Reference	
		T2	1.26 (1.05–1.50)	0.011	1.29 (1.08–1.55)	0.005
		T3	1.43 (1.20–1.70)	< 0.001	1.45 (1.21–1.74)	< 0.001
		*P*-trend		< 0.001		< 0.001
	Female	TyG index	1.22 (1.11–1.34)	< 0.001	1.23 (1.11–1.36)	< 0.001
		T1	Reference		Reference	
		T2	1.33 (1.10–1.61)	0.003	1.35 (1.10–1.65)	0.004
		T3	1.57 (1.30–1.90)	< 0.001	1.57(1.28–1.93)	< 0.001
		*P*-trend		< 0.001		< 0.001
*P*-interaction						< 0.001

T1: TyG index < 10.09, T2: 10.09 ≤ TyG index ≤ 10.71, T3: TyG index > 10.71.

a Model 1: adjusted for sex, age, if applicable.

b Model 2: adjusted for sex, age, smoking, alcohol consumption, SBP, DBP, TG, HDL-C, uric acid, urea, creatinine, hypertension, hyperlipidemia, and use of antihypertensives, antilipidemic drugs, and antiplatelets, if applicable.

TyG, triglyceride-glucose index; HFpEF, heart failure with preserved ejection fraction; OR, odds ratio; CI, confidence interval.

Regardless of sex, this association remained statistically significant before and after multivariate adjustment. After multivariate adjustment, the OR value of TyG index and HFpEF was higher in women (OR: 1.23; 95% CI: 1.11–1.36) than in men (OR: 1.17; 95% CI: 1.02–1.22). Multivariate logistic regression analysis showed that the TyG index levels for T2 and T3 were associated with an increased OR for HFpEF when T1 was used as a reference, with the highest association observed for T3 in both sexes (OR: 1.57; 95% CI: 1.28–1.93 for women and OR: 1.45; 95% CI: 1.21–1.74 for men).

### 3.4 Association between TyG index and the risk of HFpEF according to different metabolic status

The association between the TyG index and HFpEF varied with blood pressure and lipid status (**Table 4**). After multivariate adjustment, the TyG index of CHD patients was significantly associated with HFpEF for hypertension (OR: 1.27; 95% CI: 1.17–1.37) and dyslipidemia (OR: 1.15; 95% CI: 1.08–1.24). For both hypertension and dyslipidemia, using T1 as the reference, T2 and T3 were significantly related to the increased risks of HFpEF. Notably, T3 exhibited the strongest association in both hypertension (OR: 1.69; 95% CI: 1.44–1.97) and dyslipidemia (OR: 1.49; 95% CI: 1.29–1.73). This relationship remained significant even after multivariate adjustment.

**Table 4:** Association between TyG index and the risk of HFpEF according to hypertension and dyslipidemia.

Group		Variables	HFpEF			
			OR (95% CI)^a^	*P*-value	OR (95% CI)^b^	*P*-value
Hypertension	No	TyG index	0.98 (0.86–1.11)	0.728	0.95 (0.83–1.09)	0.480
		T1	Reference		Reference	
		T2	1.22 (1.94–1.59)	0.136	1.13 (0.86–1.47)	0.392
		T3	1.23 (1.94–1.59)	0.125	1.17 (1.89–1.54)	0.269
		*P*-trend		0.120		0.262
	Yes	TyG index	1.24 (1.16–1.34)	< 0.001	1.27 (1.17–1.37)	< 0.001
		T1	Reference		Reference	
		T2	1.32 (1.14–1.53)	< 0.001	1.42 (1.21–1.65)	< 0.001
		T3	1.39 (1.38–1.85)	< 0.001	1.69 (1.44–1.97)	< 0.001
		*P*-trend		< 0.001		< 0.001
*P*-interaction						0.005
Dyslipidemia	No	TyG index	1.26 (1.06–1.49)	0.008	1.27 (1.06–1.52)	0.008
		T1	Reference		Reference	
		T2	1.24 (0.93–1.65)	0.141	1.19 (0.88–1.61)	0.243
		T3	1.66 (1.22–2.26)	0.001	1.70 (1.23–2.35)	0.001
		*P*-trend		0.001		0.002
	Yes	TyG index	1.77 (1.10–1.26)	< 0.001	1.15 (1.08–1.24)	< 0.001
		T1	Reference		Reference	
		T2	1.32 (1.41–1.53)	< 0.001	1.35 (1.06–1.57)	< 0.001
		T3	1.53 (1.33–1.76)	< 0.001	1.49 (1.29–1.73)	< 0.001
		*P*-trend		< 0.001		< 0.001
*P*-interaction						< 0.001

T1: TyG index < 10.09, T2: 10.09 ≤ TyG index ≤ 10.71, T3: TyG index > 10.71.

a Model 1: adjusted for sex, and age.

b Model 2: adjusted for sex, age, smoking, alcohol consumption, SBP, DBP, TG, HDL-C, uric acid, urea, creatinine, hypertension, hyperlipidemia, and use of antihypertensives, antilipidemic drugs, and antiplatelets, if applicable.

TyG, triglyceride-glucose index; HFpEF, heart failure with preserved ejection fraction; OR, odds ratio; CI, confidence interval.

### 3.5 Association between TyG index and the risk of HFpEF according to age and metabolic status

The TyG index and HFpEF demonstrated a significant association with hypertension (OR: 1.24; 95% CI: 1.07–1.45) among CHD patients aged ≤ 60 years (**Table 5**). Using T1 as the reference, T2 and T3 were significantly related to the increased risk of HFpEF, and T3 exhibited the strongest association (OR: 1.67; 95% CI: 1.19–2.34). After multivariate adjustment, the TyG index demonstrated a similar association with HFpEF among individuals aged > 60 years, for both hypertension (OR: 1.28; 95% CI: 1.17–1.40) and dyslipidemia (OR: 1.17; 95% CI: 1.07–1.27). For both hypertension and dyslipidemia in patients aged > 60 years, using T1 as the reference, T2 and T3 were significantly related to the increased risk of HFpEF, and T3 exhibited the strongest association for both hypertension (OR: 1.71; 95% CI: 1.43–2.04) and dyslipidemia (OR: 1.49; 95% CI: 1.26–1.76). This relationship remained significant even after multivariate adjustment.

**Table 5:** Association between TyG index and the risk of HFpEF according to age and metabolic status.

Group			Variables	HFpEF			
				OR (95% CI)^a^	*P*-value	OR (95% CI)^b^	*P*-value
≤ 60	Hypertension	No	TyG index	0.77 (0.59–1.00)	0.05	0.75 (0.56–1.00)	0.049
			T1	Reference		Reference	
			T2	1.28 (0.78–2.10)	0.33	1.08 (0.64–1.82)	0.787
			T3	0.92 (0.55–1.54)	0.738	0.89 (0.51–1.56)	0.684
			*P*-trend		0.789		0.713
		Yes	TyG index	1.17 (1.02–1.34)	0.027	1.24 (1.07–1.45)	0.005
			T1	Reference		Reference	
			T2	1.30 (0.96–1.77)	0.091	1.58 (1.13–2.22)	0.008
			T3	1.36 (1.01–1.82)	0.044	1.67 (1.19–2.34)	0.003
			*P*-trend		0.051		0.004
	Dyslipidemia	No	TyG index	0.83 (0.55–1.26)	0.387	0.81 (0.52–1.28)	0.369
			T1	Reference		Reference	
			T2	0.83 (0.45–1.55)	0.562	0.71 (0.35–1.41)	0.324
			T3	0.92 (0.46–1.85)	0.816	0.79 (0.37–1.72)	0.556
			*P*-trend		0.739		0.470
		Yes	TyG index	1.11 (0.98–1.26)	0.110	1.10 (0.96–1.26)	0.157
			T1	Reference		Reference	
			T2	1.46 (1.10–1.96)	0.010	1.57 (1.15–2.15)	0.005
			T3	1.41 (1.06–1.87)	0.017	1.48 (1.09–2.01)	0.012
			*P*-trend		0.025		0.019
> 60	Hypertension	No	TyG index	1.06 (0.92–1.23)	0.424	1.03 (0.88–1.21)	0.707
			T1	Reference		Reference	
			T2	1.17 (0.86–1.60)	0.312	1.11 (0.81–1.53)	0.514
			T3	1.34 (0.99–1.81)	0.058	1.28 (0.93–1.76)	0.138
			*P*-trend		0.001		0.139
		Yes	TyG index	1.16 (1.06–1.26)	0.001	1.28 (1.17–1.40)	< 0.001
			T1	Reference		Reference	
			T2	1.33 (1.12–1.57)	0.001	1.38 (1.16–1.65)	< 0.001
			T3	1.68 (1.42–1.99)	< 0.001	1.71 (1.43–2.04)	< 0.001
			*P*-trend		< 0.001		< 0.001
	Dyslipidemia	No	TyG index	1.37 (1.13–1.65)	0.001	1.4 (1.14–1.71)	0.001
			T1	Reference		Reference	
			T2	1.33 (0.96–1.84)	0.084	1.27 (0.91–1.79)	0.162
			T3	1.89 (1.33–2.68)	< 0.001	1.97 (1.36–2.85)	< 0.001
			*P*-trend		< 0.001		< 0.001
		Yes	TyG index	1.2 (1.11–1.30)	< 0.001	1.17 (1.07–1.27)	< 0.001
			T1	Reference		Reference	
			T2	1.27 (1.08–1.51)	0.005	1.29 (1.09–1.53)	0.004
			T3	1.57 (1.34–1.85)	< 0.001	1.49 (1.26–1.76)	< 0.001
			*P*-trend		< 0.001		< 0.001

T1: TyG index < 10.09, T2: 10.09 ≤ TyG index ≤ 10.71, T3: TyG index > 10.71.

a Model 1: adjusted for sex.

b Model 2: adjusted for sex, age, smoking, alcohol consumption, SBP, DBP, TG, HDL-C, uric acid, urea, creatinine, hypertension, hyperlipidemia, use of antihypertensives, use of antilipidemic drugs, and use of antiplatelets, if applicable.

TyG, triglyceride-glucose index; HFpEF, heart failure with preserved ejection fraction; OR, odds ratio; CI, confidence interval.

### 3.6 Association between TyG index and the risk of HFpEF according to sex and metabolic status

Regardless of sex and different blood pressure and lipid statuses among CHD patients, the association between TyG index and HFpEF was consistent (**Table 6**). After multivariate adjustment, the TyG index of CHD patients was significantly associated with HFpEF for hypertension (OR: 1.23; 95% CI: 1.11–1.37) and dyslipidemia (OR: 1.15; 95% CI: 1.04–1.26) among men. Similarly, the TyG index of CHD patients was significantly associated with HFpEF among women for both hypertension (OR: 1.31; 95% CI: 1.16–1.47) and dyslipidemia (OR: 1.16; 95% CI: 1.04–1.30). After multivariate adjustment using T1 as the reference, T2 and T3 were significantly related to an increased risk of HFpEF, and T3 showed the strongest association. The association between the TyG index and HFpEF in hypertension was stronger than that in hyperlipidemia in both sexes.

**Table 6:** Association between TyG index and the risk of HFpEF according to sex and metabolic status.

Group			Variables	HFpEF			
				OR (95% CI)^a^	*P*-value	OR (95% CI)^b^	*P*-value
Male	Hypertension	No	TyG index	0.88 (0.75–1.04)	0.141	0.88 (0.74–1.05)	0.153
			T1	Reference		Reference	
			T2	1.03 (0.71–1.48)	0.89	0.97 (0.67–1.41)	0.883
			T3	1.08 (0.77–1.52)	0.667	1.08 (0.75–1.54)	0.684
			*P*-trend		0.665		0.679
		Yes	TyG index	1.24 (1.12–1.37)	< 0.001	1.23 (1.11–1.37)	< 0.001
			T1	Reference		Reference	
			T2	1.33 (1.09–1.63)	0.005	1.44 (1.17–1.78)	0.001
			T3	1.57 (1.29–1.92)	< 0.001	1.63 (1.31–2.01)	< 0.001
			*P*-trend		< 0.001		< 0.001
	Dyslipidemia	No	TyG index	1.07 (0.85–1.35)	0.573	1.09 (0.85–1.40)	0.493
			T1	Reference		Reference	
			T2	1.11 (0.74–1.64)	0.617	1.03 (0.68–1.56)	0.877
			T3	1.34 (0.88–2.05)	0.178	1.43 (0.91–2.23)	0.122
			*P*-trend		0.185		0.144
		Yes	TyG index	1.15 (1.05–1.26)	0.002	1.15 (1.04–1.26)	0.004
			T1	Reference		Reference	
			T2	1.3 (1.07–1.58)	0.01	1.38 (1.12–1.69)	0.002
			T3	1.49 (1.24–1.80)	< 0.001	1.52 (1.25–1.85)	< 0.001
			*P*-trend		< 0.001		< 0.001
Female	Hypertension	No	TyG index	1.12 (0.92–1.37)	0.248	1.03 (0.83–1.29)	0.773
			T1	Reference		Reference	
			T2	1.47 (1.01–2.14)	0.046	1.33 (0.89–1.97)	0.164
			T3	1.38 (0.93–2.05)	0.111	1.20 (0.77–1.87)	0.423
			*P*-trend		< 0.001		0.333
		Yes	TyG index	1.24 (1.12–1.39)	< 0.001	1.31 (1.16–1.47)	< 0.001
			T1	Reference		Reference	
			T2	1.29 (1.04–1.62)	0.023	1.37 (1.08–1.72)	0.009
			T3	1.61 (1.29–2.00)	< 0.001	1.72 (1.36–2.18)	< 0.001
			*P*-trend		< 0.001		< 0.001
	Dyslipidemia	No	TyG index	1.49 (1.16–1.93)	0.002	1.54 (1.18–2.02)	0.002
			T1	Reference		Reference	
			T2	1.35 (0.89–2.04)	0.159	1.43 (0.92–2.22)	0.11
			T3	2.04 (1.30–3.20)	0.002	2.06 (1.27–3.34)	0.003
			*P*-trend		< 0.001		0.003
		Yes	TyG index	1.2 (1.08–1.33)	0.001	1.16 (1.04–1.30)	0.008
			T1	Reference		Reference	
			T2	1.34 (1.08–1.66)	0.008	1.3 (1.04–1.63)	0.023
			T3	1.55 (1.26–1.91)	< 0.001	1.43 (1.14–1.78)	0.002
			*P*-trend		< 0.001		0.002

T1: TyG index < 10.09, T2: 10.09 ≤ TyG index ≤ 10.71, T3: TyG index > 10.71.

a Model 1: adjusted for age.

b Model 2: adjusted for sex, age, smoking, alcohol consumption, SBP, DBP, TG, HDL-C, uric acid, urea, creatinine, hypertension, hyperlipidemia, and use of antihypertensives, antilipidemic drugs, and antiplatelets, if applicable.

## 4 Discussion

This is the first large-scale study to demonstrate the relationship between the TyG index and HF and HFpEF in CHD patients and assess this relationship according to sex, age, and metabolic state (blood pressure and blood lipids). Overall, our data suggest that TyG index variability can increased HFpEF risk among Chinese adults.

HF is a global epidemic with an increasing prevalence, and the prognosis of patients with HF remains poor. HF is the leading cause of hospitalization in adults, with a 1-year mortality rate of 10–35%, and is considerably higher in patients with advanced HF (23). This underscores the importance of primary and secondary prevention of underlying HF conditions, including ischemic HF, management of HFpEF, and HF in older adults (24). Moreover, the number of HFpEF patients has been consistently increasing. Inadequate popularization of primary prevention has led to an increase in the number of individuals at high risk for developing HFpEF; however, continuous improvement in HFrEF treatment methods has substantially improved the LVEF of patients by more than 50%. Epidemiological data show that patients with HFpEF have all-cause and HF hospitalization rates similar to those with HFrEF. HFpEF is more common in older adults, women, and patients with hypertension (25–27). Our findings elucidated that HFpEF constituted the predominant subset among HF patients and that HFpEF patients were older, had higher systolic blood pressure, and were predominantly women, which is consistent with those of previous findings.

The TyG index and homeostasis model assessment of insulin resistance (HOMA-IR) demonstrated a close association. Moreover, the predictive value of the TyG index for IR was better than that for HOMA-IR (28). The TyG index is positively correlated with the prognosis of HF (14). The TyG index is a new biomarker of myocardial fibrosis in patients with HF and can be regarded as a useful risk stratification indicator for HF management (29). Multiple studies have indicated that a higher TyG index is associated with an increased risk of cardiovascular events, with varying degrees of risk across different populations (30–34). Therefore, further research is needed to explore the relationship with diabetic patients and HF. In study of NHANES 2007-2018, the TyG index was positively associated with the risk of HF, suggesting that the TyG index could serve as an important therapeutic target and prognostic indicator for HF (35). A high TyG index has been linked to poor prognosis in patients with HFpEF (36, 37). Additionally, the TyG index has demonstrated significant independent prognostic value regarding inpatient mortality and one-year all-cause mortality in patients with HF and chronic kidney disease (38). These studies suggest that the TyG index may play a crucial role in developing new therapeutic strategies aimed at improving the prognosis of high-risk populations with cardiovascular metabolic diseases. Notably, the TyG index is closely associated with the development of cognitive and physical impairments in individuals with insulin resistance and prediabetes (39). Furthermore, a high TyG-BMI is significantly associated with the risk of HF among participants with diabetes or prediabetes (40). These studies validate our findings that TyG has an independent association with HF in CHD patients and that there is a certain association with different types of HFpEF. Therefore, we propose that the TyG index could be considered a more convenient marker of IR and a valuable predictor of HFpEF. The discrepancies between our findings and these studies may relate to the association of the TyG index with HFpEF, as the previous cohorts were derived from the general population, whereas our study includes patients with CHD, specifically those with diabetes and prediabetes.

Established risk factors for atherosclerotic cardiovascular disease (ASCVD) include age, male sex, family history of ASCVD, obesity, hypertension, hypercholesterolemia, and diabetes mellitus (41). Therefore, the association between TyG index and HFpEF under different risk factor stratifications requires further exploration. The results of this study showed that the TyG index was independently associated with HFpEF in hypertension, dyslipidemia, and older patients (aged > 60 years). This relationship was observed in both sexes. A Shanghai-based community-based study on the relationship between macrovascular and microvascular injuries and the TyG index in older adults showed that an elevated TyG index was significantly associated with higher arterial stiffness and risk of renal microvascular injury (9). In middle-aged and older populations, an increase in the TyG index was significantly associated with hypertension and isolated systolic hypertension (42). The TyG index may represent a cost-effective and informative screening tool for metabolically obese individuals of normal weight (elevated blood pressure, hypertriglyceridemia, hyperglycemia, and low HDL cholesterol levels) (43). A high TyG index was independently associated with subclinical atherosclerosis (SA) in non-diabetic women but not in non-diabetic men. The TyG index was not associated with the presence of SA in patients with diabetes (44). Although the prevalence of coronary microvascular dysfunction among men and women with HFpEF is similar, the drivers of microvascular dysfunction may differ according to sex (45). These studies provide evidence for the difference in OR values for the relationship between the TyG index and HFpEF in this study.

In summary, the effect of TyG index in patients with cardiovascular diseases has been extensively investigated, emphasizing its potential clinical significance. Evaluation of the TyG index may have important clinical implications for risk stratification and individualized treatment of CHD patients.

## 5 Strengths and limitations

This study had certain limitations. First, because this was a multi-center study, there is a possibility of bias in the measurement methods at different research centers. However, the practitioners conducted external quality assessments between clinical laboratories in each center. Second, the retrospective design of the current study might have contributed to recall bias, and residual confounders could not be completely avoided. Therefore, any changes in the TyG index that may occur after HF treatment are unknown and require further investigation. Furthermore, the exact mechanism underlying the relationship between TyG index and HFpEF remains unclear, warranting further prospective large-scale studies.

## 6 Conclusion

This study demonstrated a significant association between the TyG index and HFpEF in CHD patients. Moreover, the association between TyG index and HFpEF in CHD patients was significantly more pronounced in patients with hypertension, dyslipidemia, and older patients aged > 60 years. In addition, the association between the TyG index and HFpEF in CHD patients showed that the OR value was higher in women than in men. The results of this study emphasize the need for a risk management strategy based on sex, age, and metabolic status to prevent the occurrence of HFpEF in CHD patients.

## Data Availability

The raw data supporting the conclusions of this article will be made available by the authors, without undue reservation.
