# Supplementary material for: Triglyceride-glucose index is associated with heart failure with preserved ejection fraction in different metabolic states in patients with coronary heart disease
Source: Front Endocrinol (Lausanne). 2024 Nov 4;15:1447072. doi: 10.3389/fendo.2024.1447072 (PMC11570926; doi:10.3389/fendo.2024.1447072)
Supplement: Supplementary file 1 [file DataSheet1.docx]

**Table S1. General characteristics of the study participants according to HF**

| Characteristic | Total (*n*=62,794) | No HF (*n*=48,799) | HF (*n*=13,995) | *P*-value |
| --- | --- | --- | --- | --- |
| Sex, n (%) |  |  |  | <0.001 |
| Male | 35031 (55.8) | 27879 (57.1) | 7152 (51.1) |  |
| Female | 27763 (44.2) | 20920(42.9) | 6843 (48.9) |  |
| Age, years, median (IQR) | 63 (57-69) | 63 (57-68) | 65 (59-70) | <0.001 |
| SBP, mmHg, median (IQR) | 135 (121-150) | 135 (121-150) | 135 (121-150) | 0.009 |
| DBP, mmHg, median (IQR) | 81 (74-90) | 81 (75-90) | 80 (73-90) | <0.001 |
| TyG index, median (IQR) | 8.83 (8.45-9.28) | 8.83 (8.45-9.27) | 8.83(8.44-9.30) | 0.142 |
| FPG, median (IQR) | 6.46 (5.36-11.95) | 5.55 (4.90-6.94) | 5.86 (5.00-7.62) | <0.001 |
| HbA1c, median (IQR) | 6.40 (5.70-7.60) | 6.50 (5.80-7.70) | 6.30 (5.70-7.30) | <0.001 |
| LDL-C, mmol/L, median (IQR) | 2.74 (2.12-3.41) | 2.77 (2.14-3.44) | 2.54 (1.96-3.22) | <0.001 |
| HDL-C, mmol/L, median (IQR) | 1.04 (0.87-1.24) | 1.06 (0.89-1.26) | 1.07 (0.91-1.28) | <0.001 |
| TG, mmol/L, median (IQR) | 1.48 (1.07-2.09) | 1.45 (1.07-2.03) | 1.41 (1.02-1.99) | <0.001 |
| TC, mmol/L, median (IQR) | 4.43 (3.69-5.23) | 4.40 (3.69-5.19) | 4.33 (3.60-5.13) | <0.001 |
| Uric Acid, μmol/L, median (IQR) | 319.1(262.3-386.0) | 316.0 (260.0-380.0) | 334.0(272-408) | <0.001 |
| Urea, μmol/L, median (IQR) | 5.40 (4.44-6.58) | 5.30 (4.40-6.40) | 5.70(4.70-7.15) | <0.001 |
| Creatinine, μmol/L, median (IQR) | 69.00 (57.90-82.00) | 69.00 (58.00-82.00) | 68.00(56.10-83.73) | <0.001 |
| Smoking, n (%) | 36939 (58.8) | 31281 (64.1) | 5658 (40.4) | <0.001 |
| Drinking, n (%) | 33226 (52.9) | 27727 (56.8) | 5499 (39.3) | <0.001 |
| Glucose regulation state, n (%) |  |  |  | <0.001 |
| Normal glucose regulation | 31051 (49.4) | 24990 (51.2) | 6061 (43.3) |  |
| Prediabetes | 14825 (23.6) | 11430 (23.4) | 3395 (24.3) |  |
| Diabetes | 16918 (27.0) | 12379 (25.4) | 4539 (32.4) |  |
| [Hypertension](javascript:;), n (%) | 46669 (74.3) | 35461 (72.7) | 11208 (80.1) | <0.001 |
| [Dyslipidemia](javascript:;), n (%) | 54571 (86.9) | 43059 (88.2) | 11512 (82.3) | <0.001 |
| Use of antihypertensives, n (%) | 47025 (74.9) | 36357 (74.5) | 10668 (76.2) | <0.001 |
| Use of antilipidemic, n (%) | 45541 (72.5) | 36006 (73.8) | 9535 (68.1) | <0.001 |
| Use of antiplatelets, n (%) | 35837 (57.0) | 29889 (61.4) | 5848 (41.7) | <0.001 |

Data are presented as median (interquartile) or number (proportion, %).

Abbreviations: TyG index: triglyceride-glucose index, SBP: systolic blood pressure, DBP: diastolic blood pressure, FPG: fasting plasma glucose, TC: total cholesterol, TG: triglycerides, HDL-C: high-density lipoprotein cholesterol, LDL-C: low-density lipoprotein cholesterol, HbA1c: glycated haemoglobin, IQR: interquartile rang.

**Table S2. General characteristics of the study participants according to the TyG index**

| Characteristic | TyG index | | | | *P*-value |
| --- | --- | --- | --- | --- | --- |
|  | Total  (*N* = 8,606) | T1  (*n* = 2,885) | T2  (*n* = 2,848) | T3  (*n* = 2,873) |  |
| Sex, n (%) |  |  |  |  | 0.293 |
| Male | 4891 (56.8) | 1629 (56.5) | 1596 (56.0) | 1666 (58.0) |  |
| Female | 3715 (43.2) | 1256 (43.5) | 1252 (44.0) | 1207 (42.0) |  |
| Age, years, median (IQR) | 64 (58–69) | 64 (58–69) | 64 (59–69) | 63 (58–69) | 0.003 |
| SBP, mmHg, median (IQR) | 140 (125–157) | 140 (124–151) | 140 (127–156) | 140 (128–160) | < 0.001 |
| DBP, mmHg, median (IQR) | 80 (80–90) | 80 (79–90) | 80 (80–90) | 80 (80–90) | 0.263 |
| TyG index, median (IQR) | 10.33 (9.98–10.98) | 9.87 (9.73–9.98) | 10.34 (10.20–10.51) | 11.35 (10.98–11.87) | < 0.001 |
| LVEF, %, median (IQR) | 62 (59–65) | 62 (59–65) | 62 (59–65) | 62 (58–65) | 0.023 |
| FPG, mmol/L, median (IQR) | 6.22 (5.20–8.59) | 4.92 (4.59–5.20) | 6.23 (5.83–6.78) | 10.34 (8.59–13.40) | < 0.001 |
| HbA1c, %, median (IQR) | 6.50 (5.70–7.96) | 5.70 (5.30–6.40) | 6.30 (5.70–7.20) | 8.10 (6.80–9.60) | < 0.001 |
| LDL-C, mmol/L, median (IQR) | 2.82 (2.17–3.52) | 2.75 (2.14–3.43) | 2.88 (2.22–3.58) | 2.85 (2.18–3.55) | < 0.001 |
| HDL-C, mmol/L, median (IQR) | 1.02 (0.85–1.24) | 1.06 (0.88–1.30) | 1.03 (0.86–1.25) | 0.96 (0.80–1.16) | < 0.001 |
| TG, mmol/L, median (IQR) | 1.38 (1.00–1.97) | 1.24 (0.90–1.70) | 1.39 (1.01–1.96) | 1.55 (1.10–2.28) | < 0.001 |
| TC, mmol/L, median (IQR) | 4.47 (3.68–5.33) | 4.43 (3.65–5.23) | 4.48 (3.71–5.35) | 4.50 (3.68–5.38) | 0.028 |
| Uric acid, μmol/L, median (IQR) | 317 (255–391) | 320.00 (258–391) | 324(262–396.25) | 309 (247–387) | <0.001 |
| Urea, μmol/L, median (IQR) | 5.63 (4.54–7.10) | 5.38 (4.39–6.72) | 5.59 (4.47–6.95) | 5.99 (4.81–7.61) | <0.001 |
| Creatinine, μmol/L, median (IQR) | 70.90 (58.60–86.70) | 71.40 (59.40–86.40) | 70.85 (59.00–86.30) | 70.30 (57.30–87.20) | 0.109 |
| Smoking, n (%) | 3088 (35.9) | 1086 (37.6) | 1008 (35.4) | 994 (34.6) | 0.044 |
| Alcohol consumption, n (%) | 7410 (86.1) | 2485 (86.1) | 2458 (86.3) | 2467 (85.9) | 0.890 |
| Type of HF, n (%) |  |  |  |  | 0.880 |
| HFrEF | 134 (1.6) | 35 (1.2) | 42 (1.5) | 57 (2.0) |  |
| HFmrEF | 240 (2.8) | 69 (2.4) | 80 (2.8) | 91 (3.2) |  |
| HFpEF | 1896 (22.0) | 531 (18.4) | 645 (22.7) | 720 (25.1) |  |
| NYHA, n (%) |  |  |  |  | 0.813 |
| I | 130 (1.5) | 42 (1.5) | 38 (1.3) | 50 (1.7) |  |
| II | 972 (11.3) | 261 (9.0) | 341 (12.0) | 370 (12.9) |  |
| III | 687 (8.0) | 189 (6.6) | 244 (8.6) | 254 (8.8) |  |
| IV | 152 (1.8) | 44 (1.5) | 54 (1.9) | 54 (1.9) |  |
| [Hypertension](javascript:;), n (%) | 6486 (75.37) | 2097 (72.69) | 2203 (77.35) | 2186 (76.09) | < 0.001 |
| [Dyslipidemia](javascript:;), n (%) | 7268 (84.45) | 2354 (81.59) | 2364 (83.01) | 2550 (88.76) | < 0.001 |
| Glucose regulation state, n (%) |  |  |  |  | < 0.001 |
| Normal glucose regulation | 3117 (36.22) | 2885 (100.00) | 232 (8.15) | 0 (0.00) |  |
| Prediabetes | 2143 (24.90) | 0 (0.00) | 2143 (75.25) | 0 (0.00) |  |
| Diabetes | 3346 (38.88) | 0 (0.00) | 473 (16.61) | 2873 (100.00) |  |
| Use of antihypertensives, n (%) | 6331 (73.6) | 2083 (72.2) | 2081 (73.1) | 2167 (75.4) | 0.016 |
| Use of antilipidemic drugs, n (%) | 5450 (63.3) | 1823 (63.2) | 1792 (62.9) | 1835 (63.9) | 0.744 |
| Use of antiplatelets, n (%) | 4564 (53.0) | 1418 (49.2) | 1510 (53.0) | 1636 (56.9) | < 0.001 |

Data are presented as median (interquartile) or number (proportion, %).

T1: TyG index < 10.09, T2: 10.09 ≤ TyG index ≤ 10.71, T3: TyG index > 10.71.

Abbreviations: TyG: triglyceride-glucose index, SBP: systolic blood pressure, DBP: diastolic blood pressure, FPG: fasting plasma glucose, TC: total cholesterol, TG: triglycerides, HDL-C: high-density lipoprotein cholesterol, LDL-C: low-density lipoprotein cholesterol, HbA1c: glycated haemoglobin, IQR: interquartile range.

**Table S3. Association between the TyG index and the risk of HF**

| Variables | HF | | | |
| --- | --- | --- | --- | --- |
|  | OR (95% CI)^a^ | *P-*value | OR (95% CI)^b^ | *P-*value |
| TyG index | 1.06 (1.03-1.09) | < 0.001 | 1.08 (1.01-1.16) | 0.022 |
| T1 | Reference |  | Reference |  |
| T2 | 0.98 (0.94-1.02) | 0.366 | 0.96 (0.89-1.04) | < 0.001 |
| T3 | 1.10 (1.05-1.15) | < 0.001 | 1.14 (1.04-1.25) | 0.006 |
| *P*-trend |  | < 0.001 |  | 0.022 |

^a^Model 1: adjusted for sex, age;

^b^Model 2: adjusted for sex, age, smoking, drinking, SBP, DBP, TG, HDL-C, uric acid, urea, creatinine, hypertension, hyperlipidemia, use of antihypertensives, use of antilipidemic, and use of antiplatelets, if applicable.

Compared with no HF.

**Table S4. Associations between univariate and the risk of HFpEF**

| Variables | HFpEF | | |
| --- | --- | --- | --- |
|  | Beta | OR (95%CI) | *P* |
| Sex |  |  |  |
| Male |  | 1.00 (Reference) |  |
| Female | 0.20 | 1.22 (1.10 - 1.35) | < 0.001 |
| Age, years | 0.03 | 1.03 (1.02 - 1.04) | < 0.001 |
| SBP, mmHg | 0.01 | 1.01 (1.01 - 1.01) | < 0.001 |
| DBP, mmHg | -0.00 | 1.00 (1.00 - 1.00) | 0.822 |
| FPG, % | 0.02 | 1.02 (1.01 - 1.04) | < 0.001 |
| HbA1c, mmol/L | -0.02 | 0.98 (0.95 - 1.01) | 0.114 |
| LDL-C, mmol/L | -0.04 | 0.96 (0.91 - 1.01) | 0.082 |
| HDL-C, mmol/L | -0.08 | 0.93 (0.79 - 1.08) | 0.343 |
| TG, mmol/L | -0.03 | 0.97 (0.94 - 1.01) | 0.191 |
| TC, mmol/L | -0.06 | 0.94 (0.90 - 0.98) | 0.002 |
| Creatinine, μmol/L | 0.00 | 1.01 (1.01 - 1.01) | < 0.001 |
| Uric Acid, μmol/L | 0.00 | 1.01 (1.01 - 1.01) | < 0.001 |
| Urea, μmol/L | 0.08 | 1.08 (1.07 - 1.09) | < 0.001 |
| LVEF, % | -0.01 | 0.99 (0.98 - 0.99) | 0.035 |
| Smoking |  |  |  |
| No |  | 1.00 (Reference) |  |
| Yes | -0.24 | 0.78 (0.70 - 0.87) | < 0.001 |
| Drinking |  |  |  |
| No |  | 1.00 (Reference) |  |
| Yes | -0.17 | 0.85 (0.73 - 0.98) | 0.022 |
| Hypertension |  |  |  |
| No |  | 1.00 (Reference) |  |
| Yes | 0.123 | 1.13 (1.10 - 1.28) | 0.046 |
| Dyslipidemia |  |  |  |
| No |  | 1.00 (Reference) |  |
| Yes | 0.317 | 1.37 (1.20 - 1.57) | < 0.001 |
| Use of antihypertensives |  |  |  |
| No |  | 1.00 (Reference) |  |
| Yes | -0.08 | 0.93 (0.83 - 1.04) | 0.199 |
| Use of antilipidemic |  |  |  |
| No |  | 1.00 (Reference) |  |
| Yes | 0.07 | 1.07 (0.97 - 1.19) | 0.190 |
| Use of statins |  |  |  |
| No |  | 1.00 (Reference) |  |
| Yes | 0.32 | 1.38 (1.24 - 1.53) | < 0.001 |
| Use of antiplatelets |  |  |  |
| No |  | 1.00 (Reference) |  |
| Yes | 0.28 | 1.32 (1.19 - 1.46) | < 0.001 |

Abbreviations: *OR:* Odds ratio, *CI*: Confidence interval, *β*: Regression coefficient.

**Table S5. Association between the TyG index and the risk of different types of heart failure**

| Variables | HFrEF | | HFmrEF | | HFpEF | |
| --- | --- | --- | --- | --- | --- | --- |
|  | OR (95% CI)^a^ | OR (95% CI)^b^ | OR (95% CI)^a^ | OR (95% CI)^b^ | OR (95% CI)^a^ | OR (95% CI)^b^ |
| TyG index | 1.23 (1.01-1.50)^*^ | 1.39 (1.12-1.72)^**^ | 1.16 (0.99-1.35) | 1.31 (1.11-1.53)^**^ | 1.18 (1.11-1.26)^**^ | 1.17 (1.09-1.25)^**^ |
| T1 | Reference | Reference | Reference | Reference | Reference | Reference |
| T2 | 0.94 (0.65-1.36) | 1.45 (0.90-2.34) | 1.02 (0.78-1.35) | 1.46 (1.03-2.07)^*^ | 1.05 (0.94-1.17) | 1.33 (1.16-1.53)^**^ |
| T3 | 1.57 (1.11-2.23)^*^ | 2.36 (1.49-3.73)^**^ | 1.32 (1.01-1.72)^*^ | 1.97 (1.39-2.77)^**^ | 1.34 (1.21-1.49)^**^ | 1.53 (1.33-1.76)^**^ |

^a^Model 1: adjusted for sex, age;

^b^Model 2: adjusted for sex, age, smoking, drinking, SBP, DBP, TG, HDL-C, uric acid, urea, creatinine, hypertension, hyperlipidemia, use of antihypertensives, use of antilipidemic, and use of antiplatelets, if applicable.

Compared with no HF, ^*^ *P* < 0.05, ^**^ *P* < 0.01.
